# Fracture of the Os Suffraginus with Complete Recovery

**Published:** 1883-10

**Authors:** R. Lovell

**Affiliations:** Potsdam, N. Y.


### II.—FRACTURE OF OS SUFFRAGINIS WITH COMPLETE RECOVERY.

BY R. LOVELL, V.S.

On January 17, 1883, I was telegraphed, "to come and see a young trotting mare that ran away and broke her leg." On examination I found the os suffraginis fractured, but no external wound.

*History of the Case.*—She had been tied at a door; got frightened; broke loose, ran away, slipped and fell, her right forward foot slipped under the stone coping of sidewalk, and her struggles to get up resulted in above fracture.

*Treatment.*—Assisted by J. Collinson, M.D., C.M., I applied a glue bandage, and, as she was in a very plethoric condition, gave her a brisk cathartic.

Aloes. Barb., - - - - - - - ʒvii<br>

Hydrargi. Subchlorid, - - - - ʒi<br>

G. Radix, Po - - - - - - - ʒii<br>

Sap. Molis, q. s. to form Ball.<br>

Sig. at once.

Emptied rectum by hand and used enema of soap and water. Ordered tinct. Aconitum Ms. xv every two hours until cathartic operated. Diet to consist of bran mashes, carrots, etc., etc. On leaving, gave instructions to keep leg perfectly quiet till bandage had thoroughly dried, as they were unwilling to go to the expense of slinging her. On Feb. 27th I was telegraphed to come and see her again, as they thought they could take her home (some ten miles from where the accident occurred.) Found her walking around the box stall to all appearance well, but did not consider it advisable to allow them to do so. She remained another week, went home and has been doing fast work since.

POTSDAM, N. Y.

